# Jasmonic Acid Pathway in Plants 2.0

**DOI:** 10.3390/ijms22073506

**Published:** 2021-03-29

**Authors:** Kenji Gomi

**Affiliations:** Plant Genome and Resource Research Center, Faculty of Agriculture, Kagawa University, Miki, Kagawa 761-0795, Japan; gomi.kenji@kagawa-u.ac.jp

The plant hormone jasmonic acid (JA) and its derivative, an amino acid conjugate of JA (jasmonoyl isoleucine: JA-Ile), are signaling compounds involved in the regulation of cellular defense and development in plants. The number of articles on JA has increased dramatically since the 1990s. JA was recognized as a stress hormone that regulates plant responses to biotic stresses as well as abiotic stresses. Recent studies have progressed remarkably in the understanding of the importance of the JA in the life cycle of plants. It has been revealed that JA is directly involved in many physiological processes, including stamen growth, senescence, and root growth. Furthermore, it has been known to regulate the production of various metabolites such as phytoalexins and terpenoids. Many active regulatory proteins involved in the JA signaling pathway have been identified by screening for *Arabidopsis thaliana* mutants. Discovery of the JA-Ile receptor, COI1, and the central repressors, JAZs further promotes the efforts to understand the JA signaling pathway in many plant species. However, many aspects about the JA signaling pathway in other plant species remain to be elucidated.

This Special Issue, “Jasmonic Acid Pathway in Plants 2.0” contains two review articles. The review by Ueda et al. [1] summarized the current status of plant chemical biology as it pertains to JAs. Recent advances in plant hormone biology, especially a vastly improved understanding of the unique coreceptor system, has enabled the development of chemical tools, including antagonists and agonists. Chemical tools have greatly contributed to our understanding and ability to control the signaling pathway of plant hormones and are needed to probe the precise mechanistic pathways by which plants develop. Ueda and colleagues are the pioneers in the study of JA-related chemical biology and have found some antagonists and agonists to regulate JA signaling [1,2]. Progress in the development of novel chemical tools will advance the tuning and regulation of JA responses in plants.

Wan et al. [3] summarized JA functions in signaling pathways that mediate responses to abiotic stresses such as cold, drought, salinity, and light. Recently, advancements in genomics, transcriptomics, and proteomics have provided more clues on such complex gene and protein interaction networks. Such tools also facilitate the study of the regulatory mechanisms of plant hormones exclusively, and of crosstalk under different environmental stress factors. The authors also summarized crosstalk between JA and other plant hormones, such as abscisic acid, ethylene, salicylic acid, gibberellin, auxin (indole-3-acetic acid, IAA), and brassinosteroids, for abiotic stress responses.

In addition, this Special Issue also contains six original research articles. Jiang et al. [4] demonstrated that methyl jasmonate (MeJA) is involved in stomatal closure in cucumber. The authors investigated the rapid response of stomatal conductance (Gs), net assimilation rate (A), and lipoxygenase (LOX) pathway-mediated volatiles and methanol emissions to varying MeJA concentrations in cucumber leaves with partly open stomata and in leaves with reduced Gs due to drought and darkness. The authors found that MeJA led to initial opening of stomata due to an osmotic shock, followed by MeJA concentration-dependent reduction in Gs, whereas A initially decreased, followed by recovery for lower MeJA concentrations and time-dependent decline for higher MeJA concentrations. Methanol and LOX emissions were elicited in a MeJA concentration-dependent manner. The authors also found that drought-dependent reduction in Gs ameliorated MeJA effects on foliage physiological characteristics, underscoring that MeJA primarily penetrates through the stomata. These findings suggest that the amount of stress volatiles released upon MeJA exposure not only depends on the amount of MeJA that has been taken up by the leaf, but also on the leaf oxidative status.

Lin et al. [5] demonstrated that a synthesized JA-Ile-macrolactone, JA-Ile-macrolactone **5b**, acts as an inducer to activate defense response against insect and fungal pathogens in tea plant. The authors first optimized the synthetic routine using mild toxic reagent isobutyl chloroformate instead of ethyl chloroformate for conjugation, and they used acetonitrile instead of ethyl alcohol for the better dissolution of p-toluenesulfonic acid to gain JA-Ile-macrolactone **5b**. JA-Ile-macrolactone **5b**-treated tea plants significantly inhibited the larvae weight gain of *Ectropis obliqua* larvae and the lesions caused by *Colletotrichum camelliae*. Furthermore, JA-Ile-macrolactone **5b** reduced the accumulation of eriodictyol 7-O-glucuronide, which was confirmed to promote the growth rate of *E. obliqua* larvae, in tea plants. JA-Ile-macrolactone **5b** will be a useful chemical tool to control defense response against pathogen attacks in tea plant.

Marasek-Ciolakowska et al. [6] investigated the relation between JA and auxin in the formation of the secondary abscission zone in *Bryophyllum calycinum*. The secondary abscission zone has been considered to be formed by some signals possessing a specific functional competence between neighboring cells. The authors found that the effects of IAA and MeJA on the formation of the secondary abscission zone were histologically similar. Comprehensive analyses of plant hormones revealed that the balance of the endogenous levels of IAA in both sides adjacent to the abscission zone was significantly disturbed when the secondary abscission formation was induced by the application of IAA. These results suggest that an auxin gradient is important in the formation of the secondary abscission zone in the internode segments of *B. calycinum*, and IAA gradient results from polar IAA transport from the application site. The authors concluded that IAA is important in the regulation of formation of the secondary abscission zone induced by MeJA.

Kurowska et al. [7] investigated the effect of exogenous MeJA on the expression profiles of *tonoplast intrinsic protein* (*TIP*) genes in barley. TIPs form the channels in cell membranes that are responsible for the precise regulation of the movement of water and other substrates between cell compartments. The authors have identified the *cis*-regulatory motifs for the MeJA-responsive genes in the promoter regions of all the *HvTIP* genes in their previous study [8]. In this manuscript, the authors demonstrated that the expression of four *HvTIP* genes (*HvTIP1;2*, *HvTIP2;2*, *HvTIP4;1* and *HvTIP4;2*), which may be involved in transport of the nitrogen compounds from the vacuole to the cytosol, were upregulated after MeJA treatment, and MeJA decreased the nitrogen content in barley. These results indicate that JA-responsive TIPs play an important role in the regulation of the movement of water and other substrates in barely.

Ma et al. [9] demonstrated that a JA-responsive BEL1-like transcription factor, GhBLH7-D06, was a negative regulator in the defense response against *Verticillium dahlia* in cotton. The authors found that silencing the expression of *GhBLH7-D06* could enhance the resistance against *Verticillium dahlia* in cotton, and the acquisition of resistance might be mainly due to the significant overexpression of genes related to the JA signaling pathway. The authors also identified a GhBLH7-D06-interacting protein, GhOFP3-D13, which is also a negative regulator in the defense response against *Verticillium dahlia*. Furthermore, the authors demonstrated that GhBLH7-D06 could bind to the promoter region of *GhPAL-A06* to suppress its expression and eventually lead to the inhibition of lignin biosynthesis. These findings indicate that the GhBLH7-D06/GhOFP3-D13 complex negatively regulates resistance against *Verticillium dahlia* by inhibiting lignin biosynthesis and JA signaling pathway in cotton.

Upadhyay et al. [10] identified nine lipoxygenase (LOX) genes in aquatic plant, *Spirodela polyrhiza*. Plant LOXs catalyze hydroperoxidation at either the C-9 (9-LOX) or C-13 (13-LOX) residue of linoleic acid or linolenic acid, which are the major fatty acids in plant membrane lipid and are necessary for the biosynthesis of JA. The authors revealed that the 13-LOX subfamily, with seven genes, predominates, while the 9-LOX subfamily is reduced to two genes, an opposite trend from known LOX families of other plant species.

I have a strong conviction that these reviews and original articles will help in understanding the crucial roles of JA in its response to the several environmental stresses and developments in plants. Finally, I would like to express my heartfelt gratitude to all of the authors and referees for their tremendous and relentless efforts in supporting this Special Issue. Without their valuable assistance, I would not have had even a glance of this timely and successful publication with its useful updates on the JA signaling pathway in plants 2.0.

## Data Availability

Not applicable.
